# Larvicidal and Nematicidal Activities of 3-Acylbarbituric Acid Analogues against Asian Tiger Mosquito, *Aedes albopictus*, and Pine Wood Nematode, *Bursaphelenchus xylophilus*

**DOI:** 10.3390/molecules22071196

**Published:** 2017-07-17

**Authors:** Seon-Mi Seo, Hyo-Rim Lee, Ji-Eun Lee, Yong-Chul Jeong, Hyung-Wook Kwon, Joon-Kwan Moon, Mark G. Moloney, Il-Kwon Park

**Affiliations:** 1 Department of Forest Sciences, College of Agriculture and Life Sciences, Seoul National University, Seoul 08826, Korea; popcon24@naver.com (S.-M.S.); kazu21@naver.com (H.-R.L.); jie0815@snu.ac.kr (J.-E.L.); 2 Research Institute of Agriculture and Life Science, College of Agriculture and Life Sciences, Seoul National University, Seoul 08826, Korea; 3 Chemistry Research Laboratory, University of Oxford, 12 Mansfield Rd, Oxford OS1 3TA, UK; ycjchem@yahoo.co.kr; 4 Division of Life Sciences and Bio-Resource and Environmental Center, College of Life Science & Bioengineering, Incheon National University, 119 Academy-ro, Yeonsu-gu, Incheon 22012, Korea; hwkwon@inu.ac.kr; 5 Convergence Research Center for Insect Vector, College of Life Science & Bioengineering, Incheon National University, 119 Academy-ro, Yeonsu-gu, Incheon 22012, Korea; 6 Department of Plant Life and Environmental Sciences, hankyong National University, 327 Jungangro, Anseong, Gyeonggi 17579, Korea; jkmoon@hknu.ac.kr

**Keywords:** barbiturates, insecticidal activity, mosquito, nematode, bioassay

## Abstract

Widespread concern for the occurrence of resistant strains, along with the avoidance of the use of highly toxic insecticides and their wide environmental dispersal, highlights the need for the development of new and safer pest control agents. Natural products provide inspiration for new chemical entities with biological activities, and their analogues are good lead compounds for the development of new pest control agents. For this purpose, we evaluated the larvicidal and nematicidal activities of 48 3-acylbarbituric acid analogues against the Asian tiger mosquito, *Aedes albopictus* and the pine wood nematode, *Bursaphelenchus xylophilus*, organisms of increasing global concern. Among the 48 3-acylbarbituric acid analogues, four compounds—**10**, **14d**, **14g** and **19b**—showed >90% larvicidal activity against *Ae. albopictus* at 10 μg/mL concentration, and one (compound **10**) showed the strongest larvicidal activity against *Ae. albopictus*, with a LC_50_ value of 0.22 μg/mL. Only compound **18** showed strong nematicidal activity against pine wood nematode. Most active compounds possessed similar physicochemical properties; thus, actives typically had ClogP values of around 1.40–1.50 and rel-PSA values of 16–17% and these similar cheminformatic characteristics reflect their similar structure. This study indicates that active 3-acylbarbituric acids analogues have potential as lead compounds for developing novel mosquito control agents.

## 1. Introduction

The Asian tiger mosquito, *Aedes albopictus* Skuse, is an important vector of dengue virus (DENV), chikungunya virus (CHIKV), and Zika virus, and has become increasingly important in public health because of its rapid and aggressive spread from its native area in East Asia [1]. Various synthetic pesticides including insect growth regulators (methoprene, novaluron, and pyriproxifen), organophosphates (temephos) and pyrethroids have been widely used to control the larvae and adult stage of *Ae. albopictus* around the world [2,3]. While these pesticides have proved to be important contributors to the management of the population of *Ae. albopictus*, they are not without environmental and human health effects [4], having been found to have undesirable effects on natural enemies and non-targeted organisms [5,6]. Moreover, the emergence of resistance in *Ae. albopictus* to larvicides, especially temephos, has been documented in several areas including Asia, Central and South America, and Europe [2,3].

Pine wilt disease—caused by the pine wood nematode, *B. xylophilus*—is a serious threat to the pine forests of several Asian and European countries [7]. This disease was first documented in Busan City in 1988, and has spread to several areas of the Korean peninsula [8]. Several kinds of control methods, including the felling and fumigation of dead pine trees with metham sodium, the aerial spraying of thiacloprid to control the insect vector, and trunk injection with synthetic nematicides, have been mainly used in Korea [9]. However, concerns relating to the use of fumigants and aerial spraying of synthetic pesticides have increased. The application of nematicides is considered to be less harmful to the environment because they can be injected directly into the pine tree trunk, and although there is also concern for the development of resistant pine wood nematode to conventional nematicides, there have been no reports so far.

Concern for environmental dispersal and the occurrence of resistant strains highlights the need for the development of new and safer type of pest control agents. Natural products provide inspiration for new chemical entities with biological activity, and their analogues are good sources as lead compounds for the development of new pest control agents [10]. For this purpose, larvicidal or nematicidal activities of natural products and their analogues against mosquito and pine wood nematode have been investigated in several previous studies [11,12,13,14,15].

In this study, we evaluated the larvicidal and nematicidal activities of 48 3-acylbarbituric acid analogues to find a new alternative for conventional pesticides; this approach was conducted because these compounds had recently been reported to have high levels of antibacterial activity, and of interest was whether this activity was translated into higher organisms [16]. In addition, we investigated the structure-activity relationships and physicochemical property–larvicidal activity relationships of barbituric acids analogues for further optimization of pest control agents.

## 2. Results and Discussion

### 2.1. Larvicidal and Nematicidal Activities of Barbituric Acids

The larvicidal and nematicidal activities of 48 barbituric acid analogues against *Ae. albopictus* and *B. xylophilus* are shown in Table 1.

Among test compounds, **10**, **14g**, **14d** and **19b** showed very strong larvicidal activity against *Ae. albopictus* at 10 μg/mL concentration. Mortalities of compounds **10**, **14g**, **14d** and **19b** were 100% and 100%, 97.5%, and 92.5%, respectively. Compounds **14f**, **14e**, **14c**, **15c**, **2b** and **21a** showed >70% mortality against *Ae. albopictus* at 10 μg/mL concentration. Other barbituric acid analogues showed moderate or only weak larvicidal activity. The larvicidal activity of barbituric acids with ≥70% mortality at 10 μg/mL was tested at lower concentrations, and compound **10** showed the strongest larvicidal activity, with LC_50_ of 0.22 μg/mL (Table 2). The LC_50_ value of temephos was 0.0093 μg/mL, which is ≈24 times better than that of compound **10**. The larvicidal activity of compound **14g** was 90% at 2.5 μg/mL concentration, but reduced to 2.5% at 1.25 μg/mL concentration. Other barbituric acid analogues showed <60% mortality at 5 μg/mL concentration. Only compound **18** showed strong nematicidal activity against pine wood nematode, which was 93.4% at 10 μg/mL concentration, but reduced to 3.5% at 5 μg/mL concentration. The nematicidal activity of other barbituric acid analogues was less than 10%. The antibacterial activity and low mammalian toxicity of 3-acylbarbituric acids has been reported in previous studies [16,17,18], while insecticidal (against *Aedes aegypti*), antifungal and antibacterial activities of 3-acylbarbituric acids, and of aminopropylidene analogues, have also been reported [19,20].

### 2.2. Physicochemical Property–Larvicidal Activity Relationships

**Figure 1:**
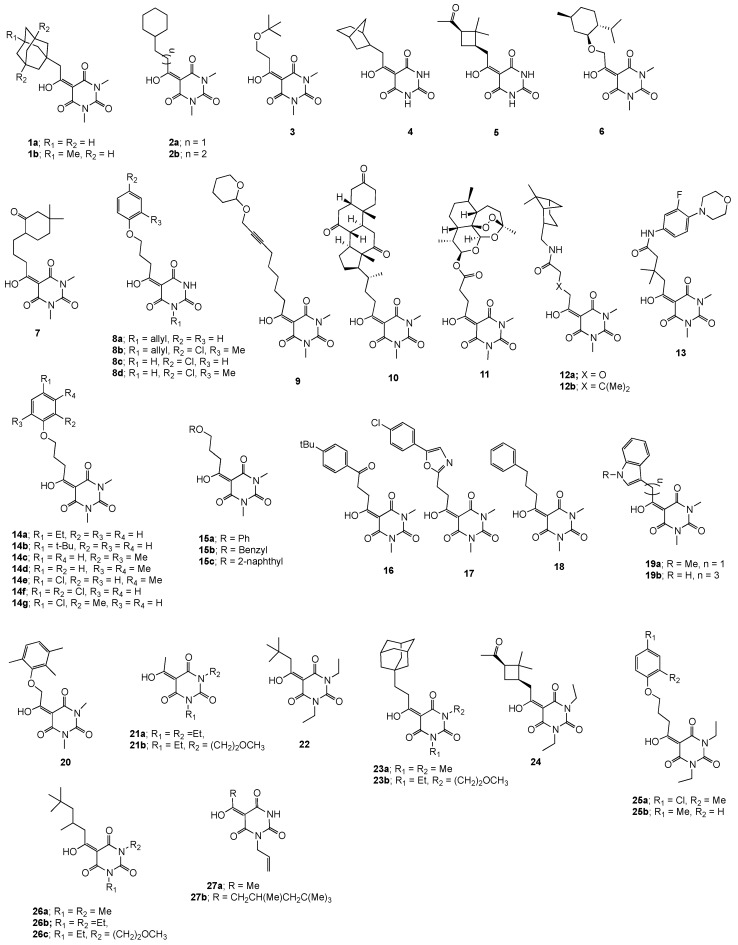
Chemical structures of 3-acylbarbituric acids analogues.

**Figure 2:**
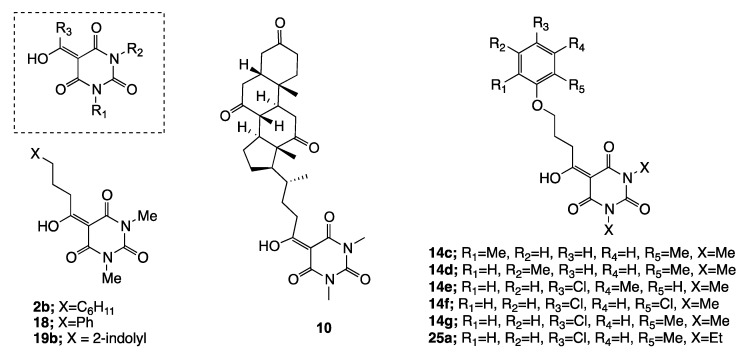
Structures with highest activity levels.

The barbiturate library, whose synthesis and characterization have been previously documented [16], comprised a series of substituted systems, varying in ring and nitrogen groups R_1_, R_2_, and R_3_ (which include a range of aliphatic and aromatic substituents, with different degrees of side chain functionality) as shown in Figure 1. Of interest is that only a small subset of the examined compounds exhibited larvicidal activity against *Ae. albopictus* (>70% mortality at 10 μg/mL), and the most active systems, shown in Figure 2, included the structurally related group—**14c**, **14d**, **14e**, **14f**, **14g**—with a disubstituted pendant aryloxypropanoyl side chain with methyl/and or chloro groups. All aliphatic side chains were inactive, with the exception of the cyclohexylpropyl system **2b**, although of interest was the high activity of the complex system **10**. By contrast, there was a much weaker spectrum of nematicidal activity against *Bursaphelenchus xylophilus,* for which only compound **18** was active, with no closely related aromatic substituted systems showing any activity. This activity pattern is of interest, because it offers the possibility of selective activity against either *Ae. Albopictus* or *Bursaphelenchus xylophilus.* With the exception of compound **10**, all active compounds possessed similar physicochemical properties (Table 3); thus, actives typically had ClogP values of around 1.40–1.50 and rel-PSA values of 16–17% and this reflects their similar structure. Of interest is that of the remaining inactive compounds, only compounds **7**, **13**, **14a**, **15c**, **16**, **20** and **24** possessed this combination of values of ClogP and rel-PSA, suggesting that compounds which are similar in structure are broadly similar in activity, and the converse. The physicochemical properties of barbituric acid analogues and antibacterial activity relationships has been discussed in a previous study [16], where barbiturates with antibacterial activity were found to possess physicochemical properties as follows: −3.0 < ClogD_7.4_ < 2.0; 0 < ClogP < 3.0; 60 < PSA < 120 Å^2^; 10 < rel-PSA < 30% and 270 < MSA < 650 Å^3^ as well as acceptable molecular weight (< 400 Da), rotatable bonds (usually less than 6), and appropriate numbers of proton-donor (1–2) and -acceptor groups (4–6).

Interestingly, 3-acylbarbituric acids with positive larvicidal or nematicidal activities possess a much narrower range of physicochemical properties. This result suggests that the physicochemical properties of active barbituric acids might provide higher permeability of compounds to the target sites not only of bacteria, but also nematodes and insects. The modes of action of structurally different larvicides or nematicides have been reported; for example, a recent study showed that the mode of action of four flavonoids and two fatty acids isolated from *Millettia pinnata* seed against three mosquito species—*Aedes aegypti*, *Ae. albopictus*, and *Culex pipens pallens*—was closely related to the acetylcholinesterase or octopaminergic systems [21]. Avermectin and emamectin benzoate, widely used to control pine wood nematode in Korea, are known to act as agonists of GABA receptors on neuromuscular cells [22]. Wang et al. [23] reported that the mode of action of Huanong AVM, an analogue of avermectin, against pine wood nematode might be related to PIP Kinase family members. However, in the work reported here, the detailed mode of action of active barbituric acids was not determined, and future work will be required to investigate this in detail.

Compound **10** is particularly interesting, because its chemical structure and physicochemical properties are different from the other active barbituric acids. It will be necessary in a future study to examine the structure–activity relationship of compound **10** and its analogues to know the essential pharmacophore for larvicidal activity against mosquito.

## 3. Materials and Methods

### 3.1. Chemicals

Chemical structures of 48 barbituric acids analogues are shown in Figure 1. Their synthesis and characterization have been previously documented [16]. Temephos (purity 95.6%) was used as a positive control and was purchased from Sigma-Aldrich (Milwaukee, WI, USA).

### 3.2. Insects

*Ae. albopictus* were reared in the laboratory, without exposure to any insecticides. *Ae. albopictus* adults were supplied with a 10% sugar solution. A live mouse was used for blood under a Korea National Institute of Health Institutional Animal Care and Use Committee (KCDC-020-11-2A) protocol approved for this study. Larvae were reared in plastic pans (24 cm × 35 cm × 5 cm) at 26 ± 1 °C, with a relative humidity of 60 ± 5%, under a 16:8 h light:dark cycle. *Ae. albopictus* larvae were supplied with sterilized diet (Super TetraMin^®^, Sewha Pet Food CO. Seoul, Korea) for food.

### 3.3.Collection of the Pine Wood Nematode

Pine wood nematode, *B. xylophilus,* was isolated from infected *Pinus densiflora* wood collected in the Haman-region, Gyeongsangnam-do province, Korea. Baermann funnel method was used to extract the pine wood nematode. The colony was maintained on a lawn of *Botrytis cinerea* cultured on potato dextrose agar medium (PDA) in the dark at 28 °C.

### 3.4. Larvicidal Activity Test

A larvicidal activity test followed the method of a previous study with slight modification [24]. Briefly, barbituric acids were serially diluted from an initial 0.01% (*w*/*v*) stock solution prepared in acetone. One mL of each compound was suspended in 200 mL of water in 270 mL paper cups. Ten early third instar larvae of *Ae. albopictus* were moved individually into a paper cup by using a glass pipette. A set of paper cups treated with 1 mL of acetone only was used as control. Treated and control larvae were kept at the same conditions used for colony maintenance, and dead and live larvae were counted 48 h after treatment. Food was not supplied to larvae during the bioassay. All treatments were replicated four times.

### 3.5. Nematicidal Activity Test

To test the nematicidal activity of barbituric acids, test compounds were dissolved in ethanol (1 mg/mL). Barbituric acid solutions (1 µL) were added to the wells of a 96-well plate (Falcon, USA) containing 50~150 nematodes (mixture of juvenile and adult nematodes, male:female:juvenile ≈ 1:1:2) in 99 µL of water. The total volume of the solution in each well was 100 µL, and the concentration of the test barbituric acids was 10 μg/mL. Four wells treated with ethanol (1 µL) only, in the same volume as the test samples, served as controls. In four adjacent wells (i.e., in a column) on the plate, nematodes were treated with barbituric acids and a set of other barbituric acids was placed in the wells of every next column. All experiments were repeated four times. The order of barbituric acids was randomly determined. The tested plates were kept in the dark at 25 ± 1 °C and 60% relative humidity. Pine wood nematode mortality was investigated after 48 h of treatment as follows: 10 µL of the test suspension was transferred to 100 µL of fresh water with a micropipette. Ten minutes after transfer, nematode mortality was observed under a microscope. Nematodes were classified as dead if their bodies were motionless and straightened.

### 3.6. Statistical Analysis

The percentages of mortality of *Ae. albopictus* mosquito larvae and pine wood nematode were transformed to arcsine square-root values prior to analysis of variance (ANOVA). Treatment mean values were compared and separated using Scheffe’s test. Mean ± SE values of untransformed data have been reported. The LC_50_ was estimated by probit analysis [25].

## 4. Conclusions

In this study, larvicidal and nematicidal activities of 48 3-acylbarbituric acids analogues against Asian tiger mosquito and pine wood nematode were evaluated. Among test compounds, compounds **10**, **14d**, **14g** and **19b** showed very strong larvicidal activities, and only compound **18** exhibited strong nematicidal activity. Structure–activity relationships study indicated that all active compounds except compound **10** possessed similar physicochemical properties. Further studies including safety of active 3-acylbarbituric acids analogues to humans and non-target organisms, formulations, and their modes of action are necessary to develop the practical use of 3-acylbarbituric acids analogues as novel mosquito-control agents.

## Figures and Tables

**Table 1:** Larvicidal and nematicidal activities of barbituric acids (see Figure 1) against *Ae. albopictus* and *B. xylophilus*

Compounds	Mortality of *Ae. albopictus* (%, Mean ± SE)				Mortality of *B. xylophilus* (%, Mean ± SE)	
	Concentration ^1^				Concentration	
	10	5	2.5	1.25	10	5
**1a**	30.0 ± 8.1bcdef ^2^	- ^3^	-	-	0.2 ± 0.2b	-
**1b**	2.5 ± 5.0f	-	-	-	1.2 ± 0.4b	-
**2a**	30.0 ± 14.1bcdef	-	-	-	1.2 ± 0.5b	-
**2b**	75.0 ± 20.8ab	0c	-	-	0.9 ± 0.4b	-
**3**	0f	-	-	-	0.2 ± 0.2b	-
**4**	2.5 ± 5.0f	-	-	-	0.3 ± 0.3b	-
**5**	0f	-	-	-	0.5 ± 0.3b	-
**6**	0f	-	-	-	0.7 ± 0.2b	-
**7**	0f	-	-	-	0.6 ± 0.3b	-
**8a**	0f	-	-	-	0.2 ± 0.2b	-
**8b**	10def	-	-	-	0.2 ± 0.2b	-
**8c**	0f	-	-	-	0.5 ± 0.5b	-
**8d**	0f	-	-	-	0.3 ± 0.3b	-
**9**	0f	-	-	-	3.4 ± 1.6b	-
**10**	100a ^2^	100a	100a	100a	0.5 ± 0.3b	-
**11**	5.0 ± 5.77f	-	-	-	0.5 ± 0.3b	-
**12a**	0f	-	-	-	0.5 ± 0.5b	-
**12b**	0f	-	-	-	0.9 ± 0.4b	-
**13**	2.5 ± 5.0f	-	-	-	0b	-
**14a**	60.0 ± 14.1abcde	2.5 ± 5.00bc	-	-	0.3 ± 0.3b	-
**14b**	12.5 ± 9.5def	-	-	-	1.4 ± 0.5b	-
**14c**	77.5 ± 15.0ab	37.5 ± 20.62bc	35.0 ± 17.32b	-	0b	-
**14d**	97.5 ± 5.0a	37.5 ± 29.36bc	-	-	0.8 ± 0.6b	-
**14e**	77.5 ± 18.9ab	15.0 ± 5.77bc	-	-	1.0 ± 0.4b	-
**14f**	87.5 ± 12.5a	40.0 ± 32.66bc	22.5 ± 15.00bc	-	0.8 ± 0.5b	-
**14g**	100a	100a	90a	2.5 ± 5.00b	0.2 ± 0.2b	-
**15a**	7.5 ± 9.5ef	-	-	-	1.6 ± 0.2b	-
**15b**	0f	-	-	-	1.3 ± 0.6b	-
**15c**	75.0 ± 20.8ab	55.0 ± 23.80ab	10c	-	0.3 ± 0.3b	-
**16**	0f	-	-	-	0.9 ± 0.5b	-
**17**	30.0 ± 16.3bcdef	-	-	-	0.9 ± 0.4b	-
**18**	50.0 ± 8.1abcdef	-	-	-	93.4 ± 2.3a	3.5 ± 0.9
**19a**	10 ± 11.5def	-	-	-	1.9 ± 1.1b	-
**19b**	92.5. ± 9.5a	10.0 ± 8.16bc	-	-	0.6 ± 0.6b	-
**20**	5.0 ± 5.7f	-	-	-	0.3 ± 0.3b	-
**21a**	77.5 ± 18.9ab	0c	-	-	0.5 ± 0.5b	-
**21b**	0f	-	-	-	0.9 ± 0.6b	-
**22**	7.5 ± 9.57cdef	-	-	-	1.3 ± 0.5b	-
**23a**	0f	-	-	-	0.4 ± 0.3b	-
**23b**	0f	-	-	-	1.2 ± 1.2b	-
**24**	0f	-	-	-	0.6 ± 0.6b	-
**25a**	70.0 ± 18.26abc	10.0 ± 11.55bc	-	-	0.5 ± 0.3b	-
**25b**	27.5 ± 15.00bcdef	-	-	-	1.7 ± 1.1b	-
**26a**	0f	-	-	-	0b	-
**26b**	62.5 ± 9.57abcd	0c	-	-	0.5 ± 0.3b	-
**26c**	17.5 ± 9.57cdef	-	-	-	0.0 ± 0.0b	-
**27a**	0f	-	-	-	0.0 ± 0.0b	-
**27b**	5.0 ± 5.7f	-	-	-	0.5 ± 0.3b	-
Control	0f	0c	0c	0b	0b	0
	F_48,147_ = 57.92, *p* < 0.0001	F_13,42_ = 21.49, *p* < 0.0001	F_5,18_ = 81.19, *p* < 0.0001	F_2,9_ = 1561.0, *p* < 0.0001	F_48,147_ = 477.83, *p* < 0.0001	

^1^ (μg/mL); ^2^ The same alphabets mean the data within a column are not different significantly (Scheffe’s test, 95%); ^3^ Not tested.

**Table 2:** LC_50_ values of compound **10** against *Ae. albopictus* larvae.

Compound	LC_50_ (μg/mL)	Regression Line	95% CI ^1^	χ^2^
**10**	0.22	y = 1.895χ + 6.253	0.17–0.29	0.547
Temephos	0.0093	y = 2.844χ + 10.850	0.0071–0.0118	3.368

^1^ Confidence interval.

**Table 3:** Physiocochemical properties of all barbiturates (see Figure 2).

Compounds	MW ^1^	MSA ^2^	PSA ^3^	%PSA ^4^	ClogP ^5^	ClogD_7.4_^6^	H-D ^7^/H-A ^8^	RB ^9^
**1a**	332	470	77.9	16.6	1.02	0.00	1/4	2
**1b**	346	506	77.9	15.4	1.45	0.46	1/4	2
**2a**	294	441	77.9	17.7	1.00	0.10	1/4	3
**2b**	308	471	77.9	16.5	1.40	0.54	1/4	4
**3**	284	432	87.2	20.2	−0.90	−2.26	1/5	4
**4**	264	343	95.5	27.8	−0.13	−1.34	3/4	2
**5**	294	406	113	27.8	−0.04	−1.28	3/5	3
**6**	352	545	87.2	16.0	1.26	−1.35	1/5	4
**7**	350	533	95.0	17.8	1.54	0.61	1/5	4
**8a**	330	441	95.9	21.7	1.00	−0.21	2/5	7
**8b**	379	489	95.9	19.6	1.98	0.48	2/5	7
**8c**	325	392	105	26.8	0.53	−1.24	3/5	5
**8d**	339	424	105	24.8	0.99	−0.63	3/5	5
**9**	392	584	96.4	16.5	1.05	−0.06	1/6	9
**10**	541	803	129	16.1	3.19	2.10	1/7	4
**11**	523	- ^10^	141	- ^9^	1.71	0.17	1/9	5
**12a**	407	599	116	19.4	−0.44	−3.42	2/6	6
**12b**	434	678	107	15.8	1.32	0.15	2/5	6
**13**	476	673	119	17.7	1.12	−0.34	2/7	6
**14a**	346	503	87.2	17.3	1.36	0.15	1/5	6
**14b**	374	569	87.2	15.3	2.13	0.91	1/5	6
**14c**	346	504	87.2	17.3	1.44	0.41	1/5	5
**14d**	346	505	87.2	17.3	1.44	0.40	1/5	5
**14e**	367	488	87.2	17.9	1.49	0.03	1/5	5
**14f**	387	473	87.2	18.4	1.54	−0.32	1/5	5
**14g**	367	489	87.2	17.8	1.49	0.03	1/5	5
**15a**	318	440	87.2	19.8	0.50	−0.65	1/5	5
**15b**	332	471	87.2	18.5	0.50	−0.59	1/5	6
**15c**	368	504	87.2	17.3	1.50	0.37	1/5	5
**16**	372	549	95.0	17.3	1.51	0.05	1/5	5
**17**	390	483	104	21.5	0.70	−0.82	1/5	4
**18**	302	424	77.9	18.4	1.20	0.27	1/4	4
**19a**	327	437	82.9	19.0	0.76	−0.63	1/4	2
**19b**	341	464	93.7	20.2	1.30	0.42	2/4	4
**20**	332	475	87.2	18.4	1.40	−1.46	1/5	3
**21a**	226	320	77.9	24.3	−0.59	−1.44	1/4	2
**21b**	256	367	87.2	23.8	−1.10	−1.99	1/5	4
**22**	282	447	77.9	17.4	1.04	0.11	1/4	4
**23a**	346	501	77.9	15.5	1.42	0.51	1/4	3
**23b**	404	613	87.2	14.2	1.60	0.70	1/5	7
**24**	350	536	95.0	17.7	1.14	0.15	1/5	5
**25a**	395	553	87.2	15.8	2.17	0.80	1/5	7
**25b**	360	537	87.2	16.2	1.65	0.53	1/5	7
**26a**	296	474	77.9	16.4	1.48	0.62	1/4	4
**26b**	324	538	77.9	14.5	2.16	1.36	1/4	6
**26c**	354	585	87.2	14.9	1.65	0.81	1/5	8
**27a**	210	256	86.7	33.9	−0.78	−1.80	2/4	2
**27b**	308	473	86.7	18.3	1.97	1.05	2/4	6

^1^ MW: molecular weight; ^2^ MSA: molecular surface area; ^3^ PSA: polar surface area; ^4^ %PSA: relative polar surface area = (PSA/MSA) × 100; ^5^ ClogP: calculated partition coefficient; ^6^ ClogD_7.4_: calculated distribution coefficient at pH 7.4; ^7^ HD: hydrogen bond donor count; ^8^ HA: hydrogen bond acceptor count; ^9^ RB: rotatable bond count; ^10^ could not be calculated.
